# CRISPR‐Cas12a enables efficient biallelic gene targeting in rice

**DOI:** 10.1111/pbi.13295

**Published:** 2019-11-27

**Authors:** Shaoya Li, Yingxiao Zhang, Lanqin Xia, Yiping Qi

**Affiliations:** ^1^ Institute of Crop Sciences (ICS) Chinese Academy of Agricultural Sciences (CAAS) Beijing China; ^2^ Department of Plant Science and Landscape Architecture University of Maryland College Park MD USA; ^3^ Institute for Bioscience and Biotechnology Research University of Maryland Rockville MD USA

**Keywords:** Cas12a, rice, gene targeting, homology‐directed repair

Dear Editor,

Harnessing genetic diversity and introducing elite alleles into commercial cultivars have been a major goal in crop breeding programmes. Gene targeting (GT) based on the homology‐directed repair (HDR) pathway is hence a holy grail in crop breeding. Although HDR has been documented by using sequence‐specific nucleases such as zinc finger nucleases (ZFNs) (Qi *et al.*, 2013) transcription activator‐like effector nucleases (TALENs) (Zhang *et al.*, 2013) and CRISPR‐Cas9 (Schiml *et al.*, 2014; Sun *et al.*, 2016) HDR events are often very rare in plants. The predominant DNA double‐strand break (DSB) repair pathway is still nonhomologous end joining (NHEJ) resulting in insertions and deletions (indels). Cas12a is a Class 2 Type V‐A system that generates staggered DNA DSBs distal to the protospacer adjacent motif (PAM). Since Cas12a generates staggered DSBs away from the seed region it may promote repeated cleavage and extensive end processing hence promoting GT. Monoallelic GT was previously reported with Cas12a in rice (Begemann *et al.*, 2017). We have recently explored LbCas12a GT at the rice *acetolactate synthase gene* *OsALS* (Li *et al.*, 2018). Initially we only obtained the monoallelic and mosaic recombinants at a low frequency (Li *et al.*, 2018). We realized our earlier LbCas12a GT system may have suffered from low nuclease activity possibly due to the choice of Cas12a low expression of CRISPR RNAs (crRNAs) or intrinsic low activity of crRNAs.

To improve Cas12a‐based GT we attempted to target *OsALS* with a new design. We chose two new crRNA target sites which are positioned outside the coding sequences of the two targeted amino acids (W548 and S627) and about 484 bp apart (Figure 1a). The use of two crRNAs could secure our success in case that one crRNA failed to cut the target site. To prevent recleavage of the Cas12a target sites after homologous recombination four synonymous mutations were introduced into each protospacer within the donor repair template (DRT) (Figure 1a). In addition two restriction sites present in the wild‐type (WT) DNA within the GT region *Xho*I and *EcoR*V were both eliminated by synonymous mutations. A 196‐bp left homology arm and a 74‐bp right homology arm were used resulting in a DRT of ~800 bp in length (Figure 1a). We developed a streamlined assembly system for generating an all‐in‐one expression vector either for *Agrobacterium*‐mediated transformation or for biolistic delivery (Figure 1b) which was based on our multiplexing CRISPR system and a high‐activity rice codon‐optimized LbCas12a (Lowder *et al.*, 2015; Tang *et al.*, 2017). First crRNA1 and crRNA2 were cloned into pYPQ131C‐RZ‐Lb (Addgene #134347) and pYPQ132D‐RZ‐Lb (Addgene #134348) which contain OsU6 and OsU3 promoters respectively. In parallel the donor was cloned into pYPQ133C (Addgene #69286). Second Golden Gate cloning was used to assemble crRNA1 crRNA2 and the DRT into pYPQ143 (Addgene # 69295). Finally a three‐way Gateway reaction was conducted to combine the assembled pYPQ143 vector with the rice codon‐optimized LbCas12a carried by pYPQ230 (Addgene #86210). The destination vector is pYPQ203 (Addgene #86207) that contains pZmUbi1 promoter for LbCas12a expression. 

**Figure 1:**
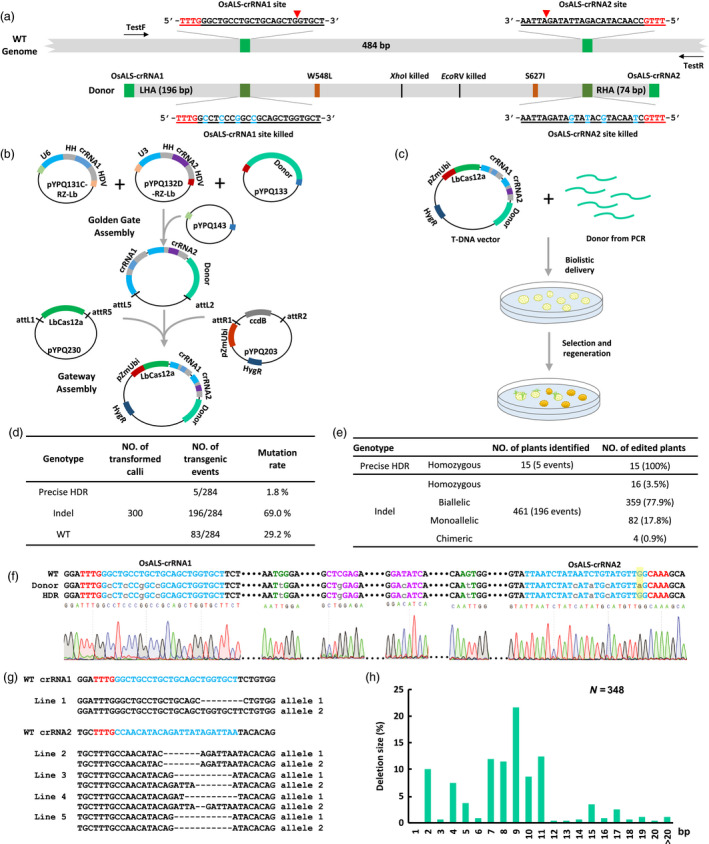
Cas12a‐based biallelic gene targeting in rice. (a) Illustration of the *OsALS* target gene, crRNA sequences and donor design. The Cas12a target sequences are underlined with protospacer adjacent motif (PAM) highlighted in red. Both target sites are indicated by light green blocks, and their modified sequences in the donor repair template (DRT) are indicated by dark green blocks. The approximate cleavage site is indicated by the red arrow. The primers used for genotyping are TestF and TestR. TestF: 5’‐GAGTTGGACCAGCAGAAGAGG‐3’. TestR: 5’‐ATGCCTACAGAAAACAACACACTACA‐3’. (b) Illustration of vectors and cloning steps for generation of the gene targeting (GT) vector. Note all the vectors used are available at Addgene. (c) Illustration of biolistic codelivery of the GT vector and additional free DRT followed by plant regeneration and selection. (d) Summary of genotyping results of all T_0_ events analysed. Note the five GT events were from calli 17, 18, 19, 20 and 21. (e) Summary of genotyping results of all edited T_0_ lines with further breakdown of insertion and deletion (Indel) mutants into different categories. Note 15 T_0_ GT lines were obtained from the five GT events: two from callus 17 (17‐1, 17‐2), three from callus 18 (18‐1, 18‐2, 18‐3), seven from callus 19 (19‐1, 19‐2, 19‐3, 19‐4, 19‐5, 19‐6, 19‐7), two from callus 20 (20‐1, 20‐2) and one from callus 21. (f) Genotyping result of a representative homozygous GT line showing biallelic precise modification based on HDR. All other GT lines have the exact same edited genotype. Note that only the complementary strand of the crRNA2 target site is shown. The lower‐case DNA letters indicate the synonymous mutations introduced into the protospacers (in blue), targeted amino acid codons for W548L and S627I (in green) and changes of two restriction enzyme sites (in purple). (g) Examples of genotyping results for mutants created by crRNA1 (only one line obtained) and crRNA2 (only four homozygous or biallelic lines are shown here). (h) A profile of deletions based on size. The result was based on analysis of 185 T_0_ plants including 6 homozygous, 157 biallelic (nonhomozygous) and 22 monoallelic plants. The total allele number (n) is 348.

Limited DRT availability in plant cells is one of the major barriers in achieving HDR events and it has been demonstrated that codelivery of the all‐in‐one vector and additional free DRT fragments enabled efficient precise gene replacement in rice (Sun *et al.*, 2016). We adopted a similar approach by codelivering the all‐in‐one vector and the free linear DNA donor into 300 rice (Japonica *cv. Nipponbare*) calli with a molar ratio of 1:20 by particle bombardment. Afterwards the calli were selected on media containing 50 mg/L hygromycin for 2–3 weeks then transferred to media containing 0.4 μm BS (bispyribac sodium) and 50 mg/L hygromycin for another 2‐3 weeks and finally regenerated on media containing 0.4 μm BS for 2–3 weeks (Figure 1c). Treating the regenerated plants from each callus as a single event we obtained a total of 284 independent events. The high regeneration rate (284 out of 300 calli) suggested the use of 0.4 μM BS posed little selection pressure for herbicide resistance consistent with our previous reports (Li *et al.*, 2018; Sun *et al.*, 2016). The genotype of each event was determined by Sanger sequencing of the PCR amplicons followed by decoding (Figure 1d). Totally we detected five precise GT events (1.8%; 5/284) while most of other events 196 out of 284 (69%) were NHEJ indels and the remainders were wild type (83 out of 284; 29.2%) (Figure 1d). We then genotyped all 476 T_0_ lines including 15 T_0_ lines from 5 HDR events and 461 T_0_ lines from 196 NHEJ events (Figure 1e). Strikingly all 15 T_0_ HDR lines were homozygous indicative of biallelic precise GT (Figure 1e). Further analysis indicated that all GT lines carried precise changes including the W548L and S627I mutations as well as mutated *Xho*I and *EcoR*V sites (Figure 1f). Our results suggested that we achieved efficient biallelic GT in rice within one generation.

Since we have included four synonymous mutations into each of the two target sites as single nucleotide polymorphisms (SNPs) probing such SNPs in GT lines may help us understand the possible mechanism of Cas12a‐mediated HDR. All four SNPs corresponding to the target site of crRNA1 were incorporated in each GT line (Figure 1f). Interestingly only three SNPs corresponding to the target site of crRNA2 were incorporated. The fourth SNP a C:G to T:A change that situated only 2 bp from the PAM was not introduced to the target site by GT (Figure 1f). To investigate the cause we analysed NHEJ events among all non‐GT lines. NHEJ‐based indels were predominantly at the crRNA2 target site: 16 out of 461 lines (3.5%) were homozygous mutants; 359 lines (77.9%) were biallelic mutants; 82 lines (17.8%) were monoallelic mutants; and 4 lines were chimeric (0.9%) (Figure 1e). By contrast only one monoallelic mutation was induced by crRNA1 (Figure 1g). This may be due to the fact that acetolactate synthase is a key enzyme for the biosynthesis of essential branched‐chain amino acids. OsALS‐crRNA1 targets the coding sequence and the resulting mutations are likely lethal. By contrast OsALS‐crRNA2 cleaves after the stop codon and the resulting mutations can be largely tolerated. It is also worth mentioning that most mutations generated by crRNA2 were large deletions (Figure 1g) which were demonstrated by profiling 348 deletion alleles out of 185 lines (Figure 1h). Consistent with our previous report (Tang *et al.*, 2017) deletions mainly occurred around the Cas12a cleavage site distal to the PAM rarely affecting the nucleotide position where we intended to introduce the fourth SNP by GT (Figure 1f). Efficient GT in our study is consistent with the synthesis‐dependent strand‐annealing (SDSA) HDR model (Puchta, 2005) and our recent analysis of Cas12a‐mediated GT in rice (Li *et al.*, 2018). Due to the nature of biolistic delivery the identified HDR lines may contain random integration of the transgene and/or the DRT fragment which however could be genetically segregated from the edited locus in the next generation.

In conclusion we here report a streamlined Cas12a system for efficient biallelic GT in rice within one generation. This study further sheds light on the SDSA mechanism of Cas12a‐mediated HDR (Li *et al.*, 2018) suggesting precise DNA sequence changes should be designed near or downstream of the cleavage site away from the nearest homology arm that is extensively used in strand invasion during SDSA. Our study also reinforces the recent demonstration of Cas12a‐mediated GT in *Arabidopsis* (Wolter and Puchta, 2019) suggesting that Cas12a is a promising tool for achieving precise gene replacement in plants 

## Author contributions

Y.Q. and L.X. conceived the study. Y.Q., Y.Z. and L.X. designed the experiments. S.L. and Y.Z. performed the experiments. Y.Q, Y.Z, S.L and L.X analysed the data and wrote the paper.

## Conflict of interests

The authors declare no competing financial interests.
